# Evidence for DNA Cleavage Caused Directly by a transfer RNA-Targeting Toxin

**DOI:** 10.1371/journal.pone.0075512

**Published:** 2013-09-17

**Authors:** Megumi Shigematsu, Tetsuhiro Ogawa, Wataru Tanaka, Kazutoshi Takahashi, Hiroko K. Kitamoto, Makoto Hidaka, Haruhiko Masaki

**Affiliations:** 1 Department of Biotechnology, The University of Tokyo, Tokyo, Japan; 2 National Institute for Agro-Environmental Sciences, Ibaraki, Japan; The Scripps Research Institute, United States of America

## Abstract

The killer yeast species *Pichia* *acaciae* produces a heteromeric killer protein, PaT, that causes DNA damage and arrests the cell cycle of sensitive *Saccharomyces cerevisiae* in the S phase. However, the mechanism by which DNA damage occurs remains elusive. A previous study has indicated that Orf2p, a subunit of PaT, specifically cleaves an anticodon loop of an *S. cerevisiae* transfer RNA (tRNA^Gln^_mcm5s2UUG_). This finding raised a question about whether the DNA damage is a result of the tRNA cleavage or whether Orf2p directly associates with and cleaves the genomic DNA of sensitive yeast cells. We showed that Orf2p cleaves genomic DNA in addition to cleaving tRNA *in vitro*. This DNA cleavage requires the same Orf2p residue as that needed for tRNA cleavage, His299. The expression of Orf2p, in which His299 was substituted to alanine, abolished the cell cycle arrest of the host cell. Moreover, the translation impairment induced by tRNA cleavage enabled Orf2p to enter the nucleus, thereby inducing histone phosphorylation.

## Introduction

Because bacteria often compete for the same niche, they produce toxins. The modes of action of these toxins are so diverse that their study sometimes leads to unexpected and intriguing observations. One example is the pore-forming activity of a large group of protein toxins such as colicins A, E1, and Ia [1]. Colicins are toxins encoded by Col-plasmids; they kill sensitive *Escherichia coli* strains that lack the cognate plasmid. Among the pore-forming colicins, a well-defined crystal structure was first described for colicin A [2]. The structure of colicin A was also found to resemble Bcl-x_L_, which belongs to the Bcl-2 family and is a regulator of programmed cell death [3]. This finding notably led to the discovery of the pore-forming activity of Bcl-x_L_ [4]. The other examples are colicin E5 and colicin D, which cleave the anticodon loops of specific transfer RNAs (tRNAs). Colicin E5 recognizes the QpUp (Q, queuosine, is a modified nucleoside of G) sequence and mediates tRNA cleavage between positions 34 and 35 in tRNA^Tyr^, tRNA^His^, tRNA^Asn^, and tRNA^Asp^ in sensitive *E. coli* cells [5,6]. Similarly, colicin D targets 4 iso-accepting tRNA ^Arg^s and cleaves between positions 38 and 39 [7].

tRNA-targeting toxins are not prokaryote-specific and are found in eukaryotes such as yeast cells. Zymocin, produced by *Kluyveromyces* *lactis* , is a heterotrimeric protein composed of α-, β- and γ-subunits [8]. The γ-subunit specifically cleaves tRNA^Glu^_mcm5s2UUC_, tRNA^Lys^_mcm5s2UUU_, and tRNA^Gln^_mcm5s2UUG_ (mcm^5^s^2^U: 5-methoxycarbonylmethyl-2-thiouridine) [9]. In sensitive *Saccharomyces cerevisiae* cells, zymoin causes cell cycle arrest in the G1 phase [10]. On the other hand, *Pichia* *acaciae* secretes a heteromeric toxin PaT, and its toxic subunit, Orf2p, cleaves tRNA^Gln^_mcm5s2UUG_ [11]. Although PaT targets specific tRNA, similar to the mode of action of zymocin, it arrests the cell cycle in the S phase, and the cell cycle arrest may presumably be mediated by Rad53p checkpoint phosphorylation [12,13]. A previous study has shown that PaT induces DNA fragmentation *in vivo*; however, the mechanism by which DNA damage is induced has been yet to be determined [14]. Several reports have shown that yeast strains with defective DNA damage repair systems exhibit different sensitivities to zymocin and PaT. For example, Rad52p plays a central role in homologous recombination, and *S. cerevisiae* lacking *RAD52* is highly sensitive to both killer toxins [11,14]. In addition, mutated alleles such as *RAD51*, *RAD55*, and *RAD59* in the *RAD52* epistasis group confer more sensitivity to PaT. Double mutation of genes encoding the apurinic/apyrimidinic endonucleases, Apn1p and Apn2p, which are responsible for base excision repair, enhance the sensitivity to zymocin and PaT [14,15]. These findings suggest that tRNA cleavage induces DNA damage. However, when D-CRD, an active domain of colicin D, was ectopically expressed in yeast cells with genetic defects in the DNA repair system, none of the cells displayed this altered sensitivity (data not shown). Although the tRNA species cleaved by D-CRD are different from those cleaved by zyomcin and PaT, this result suggested that DNA damage is not an indirect consequence of tRNA cleavage, and we tried to elucidate the mechanism by which PaT Orf2p induces DNA damage. We then found evidence that PaT Orf2p cleaves DNA, in addition to its specific tRNA cleavage activity. Moreover, the translation impairment caused by tRNA cleavage allows translocation of Orf2p into the nucleus, leading to histone phosphorylation. 

## Materials and Methods

### Yeast strains, media, and culture conditions

The *S. cerevisiae* strain CG379 (*MAT*α *ade5 can1 leu2 trp1 ura3* his7 GAL+) carrying genes for PaT Orf2p, the zymocin γ-subunit, or D-CRD integrated into the chromosome was used to express these toxins [16]. The *S. cerevisiae* strain TM142 (*MAT*α *ura3 leu2 trp1 his3*) was used for the preparation of total RNA, genomic DNA, toxin challenge, and the expression of wild-type Orf2p and its mutants by plasmid pGML20. Synthetic dextrose (SD) and synthetic galactose (SG) media were used as a synthetic minimum medium containing glucose and galactose, respectively.

Site-directed mutagenesis was performed using pGML20 carrying a gene for the C-terminal FLAG-tagged Orf2p as a template, and the resultant plasmids were introduced to yeast strain TM142. These transformants were used for the identification of the catalytic residue of Orf2p. The Orf2p-H299A-coding gene was re-cloned into pAUR101 (TaKaRa Bio, Tokyo, Japan) and introduced to a chromosome of strain CG379 as described previously [16]. Plasmid construction has been described elsewhere [16].

### Detection of histone H2A phosphorylation

Phosphorylated histone H2A was detected with western blotting using an anti-histone H2A (phospho S129) antibody (Abcam, Cambridge, UK) as described previously [14], and an anti-histone H2A antibody (BioLegend, San Diego, CA, USA) was used to detect unphosphorylated histone H2A, as a control. Both antibodies were used at a final concentration of 0.2 µg/mL, and signal detection was carried out with ECL Plus (GE Healthcare, Buckinghamshire, UK).

### Fluorescence-activated cell sorting (FACS) analysis

After the expression of toxins, cells were harvested, washed with phosphate-buffered saline (PBS), and fixed with 70% ethanol overnight. Aggregated cells were re-suspended in PBS and separated via sonication. Cells were treated with 0.5 mg/mL RNase A at 30°C overnight, and then propidium iodide was added at a final concentration of 0.025 mg/mL, followed by incubation at 4°C for 10 min. Cells were re-suspended in PBS and used for FACS analysis. Five thousand events were counted for a single acquisition and nuclear fluorescence is shown in the histogram format. The y- and x-axes are represented in a linear mode.

### Isolation of nuclei

Nuclei isolation was performed according to a previous report [17] with a minor modification. The nuclear fraction at the final step was collected via ultracentrifugation at 28,000 × *g* at 4°C for 30 min (TLA-100.3 fixed angle rotor, Beckman Coulter, Inc., Indianapolis, IN, USA) and then analyzed by western blotting with an anti-FLAG M2 antibody (Sigma, Tokyo, Japan) at a final concentration of 1 µg/mL for detection of FLAG-tagged Orf2p. To verify the fractionation, we used anti-Pgk1 (Abcam) and anti-acetyl-histone H4 (Lys16) (Upstate Biotechnology, Inc., Lake Placid, NY, USA) antibodies at concentrations of 0.6 µg/mL and 0.1 µg/mL, respectively.

### Purification of recombinant His-tagged Orf2p and the γ-subunit

The Orf2p-coding gene was amplified with polymerase chain reaction (PCR) to eliminate the coding region of the N-terminal 13-amino acid signal peptide and cloned into the *Nco*I-*Xho*I site of pET-24d (+) (Novagen, Darmstadt, Germany). The resultant plasmid expresses C-terminal His-tagged Orf2p. The plasmid was introduced to *E. coli* BL21 (DE3) (Novagen), and the cells were cultivated at 25°C. When the optical density at 660 nm reached 0.4, isopropyl-beta-d-thiogalactopyranoside was added at a final concentration of 1 mM and cultivation continued for 8 h. The cells were collected and sonificated, and the supernatant was applied to a nickel chloride-charged HiTrap Chelating HP (GE Healthcare), and Orf2p was eluted with 150 mM imidazole. For overexpression of the γ-subunit, *ORF4* of plasmid pGKL1 that encodes the γ-subunit was amplified with PCR and cloned into the *Nco*I-*Eco*RI site of pET-24d (+). The resultant plasmid encoded a C-terminal His-tagged γ-subunit lacking the signal peptide. *E. coli* BL21 (DE3) (Novagen) harboring pSTV28-IleX-ArgU encoding tRNA^Ile^_CAU_ and tRNA^Arg^_UCU_ was used as a host cell. The purification method for the γ-subunit was the same as that for Orf2p, except for elution with 135 mM imidazole. Purified proteins were dialyzed against 20 mM Tris–HCl (pH 7.5).

### *In vitro* cleavage assay

To assess tRNA cleavage, we incubated approximately 18 µg of yeast total RNA with either the Orf2 or γ-subunit for 20 min at 30°C. Then electrophoresis was carried out with 10% denaturing polyacrylamide gel electrophoresis, and specific tRNAs were detected with northern hybridization [7]. Probes for tRNA^Gln^_mcm5s2UUG_ and tRNA^Glu^_mcm5s2UUC_ have been described previously [16]. The reaction mixture used for the Orf2p assay contained 20 mM Tris–HCl (pH 7.5), 10 mM MgCl_2_, 100 mM NaCl, and 2 mM dithiothreitol. For the γ-subunit assay, BSA was also added at a final concentration of 100 µg/mL.

For the evaluation of the Orf2p activity with DNA as the substrate, approximately 300 ng of yeast genomic DNA or *Sal*I-digested pGMH10 was used. Assays were carried out under the conditions described above. After incubation, samples were separated on a 0.8% agarose gel and visualized with ethidium bromide. To exclude the Orf2p protein, we performed phenol/chloroform treatment before electrophoresis.

## Results

### tRNA cleavage dose not commonly induce DNA damage

**Figure 1:**
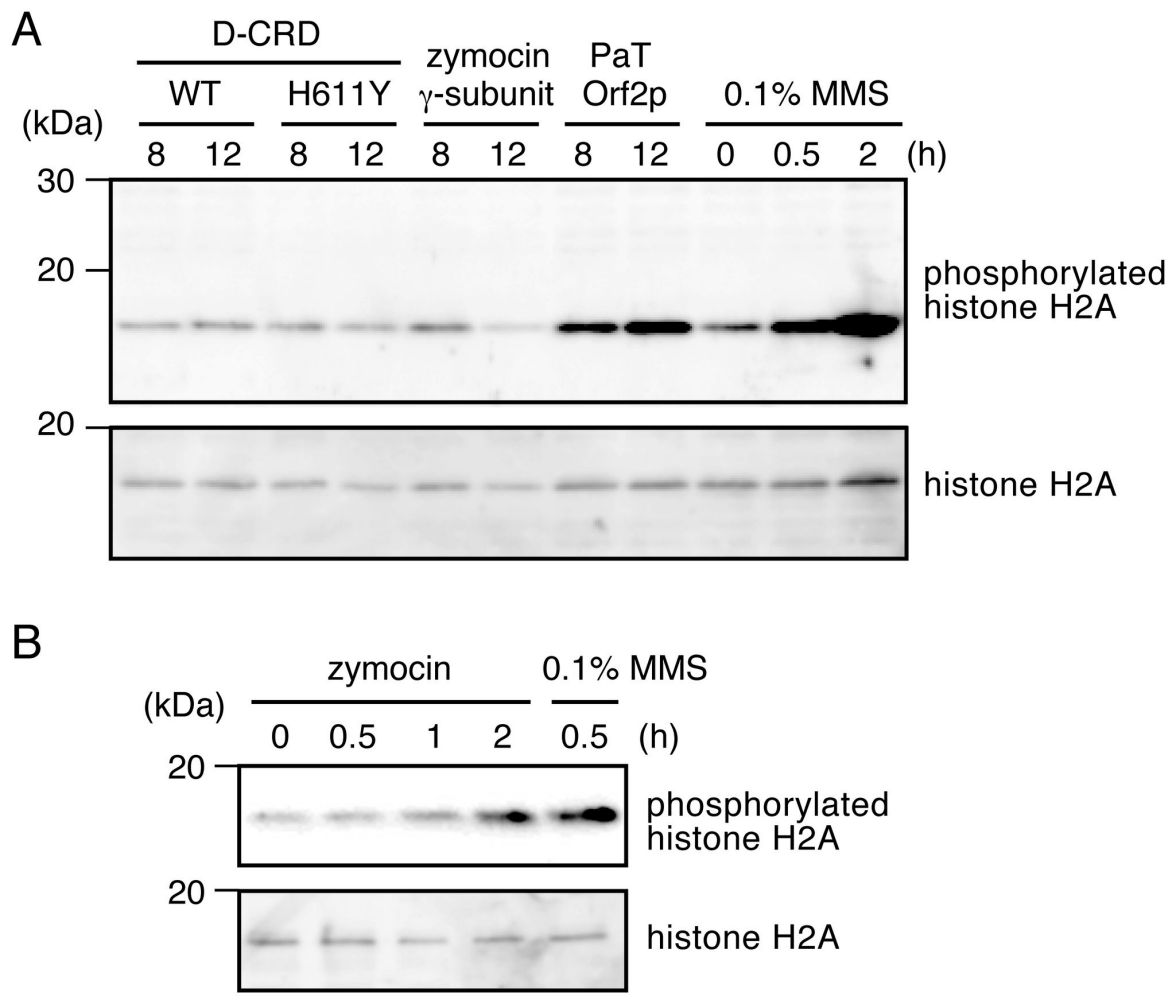
Orf2p induces histone phosphorylation. (A) Transfer RNA (tRNA)-targeting toxins were chromosomally expressed in CG379 cells, and the status of histone H2A phosphorylation was compared using western blotting. Pronounced histone phosphorylation was observed in cells expressing Orf2p. MagicMark XP (Invitrogen) was used as a molecular weight marker. (B) TM142 cells were challenged with zymocin, and phosphorylation of histone was analyzed as in (A). Histone was phosphorylated in cells challenged with zymocin for 2 hours. Methylmethane sulfonate (MMS) was used to induce DNA damage as a control.

First, we verified that DNA damage does not usually occur as a response to tRNA cleavage. DNA damage was monitored by determining the phosphorylation status of the Ser129 residue of histone H2A in cells chromosomally expressing the γ-subunit, Orf2p, or D-CRD; tRNA cleavage was previously shown to occur in the cells after the expression of these toxins [16,18]. Phosphorylation of the histone H2A Ser129 residue is a hallmark of the site of DNA damage, to which DNA repair proteins are recruited, and is one of the earliest responses after DNA damage [19,20]. The intracellular expression of the γ-subunit and D-CRD did not induce histone H2A phosphorylation (Figure 1A). In contrast, histone H2A was phosphorylated in the cells expressing Orf2p. Notably, D-CRD potentially cleaves yeast tRNA^Gln^_mcm5s2UUG_, which is the primary target of Orf2p (data not shown). These results indicate that tRNA cleavage itself does not necessarily induce DNA damage and that only Orf2p is involved in the DNA damage response. Meanwhile, histone phosphorylation in cells challenged by extracellular zymocin was also examined. After 2 hours of incubation with zymocin, low levels of histone phosphorylation were detected (Figure 1B), indicating that prolonged exposure to zymocin elicits a DNA damage response. However, based on these results, we verified that DNA damage is not a direct consequence of tRNA cleavage by zymocin.

### The His299 residue of Orf2p is responsible for DNA damage as well as tRNA cleavage

**Figure 2:**
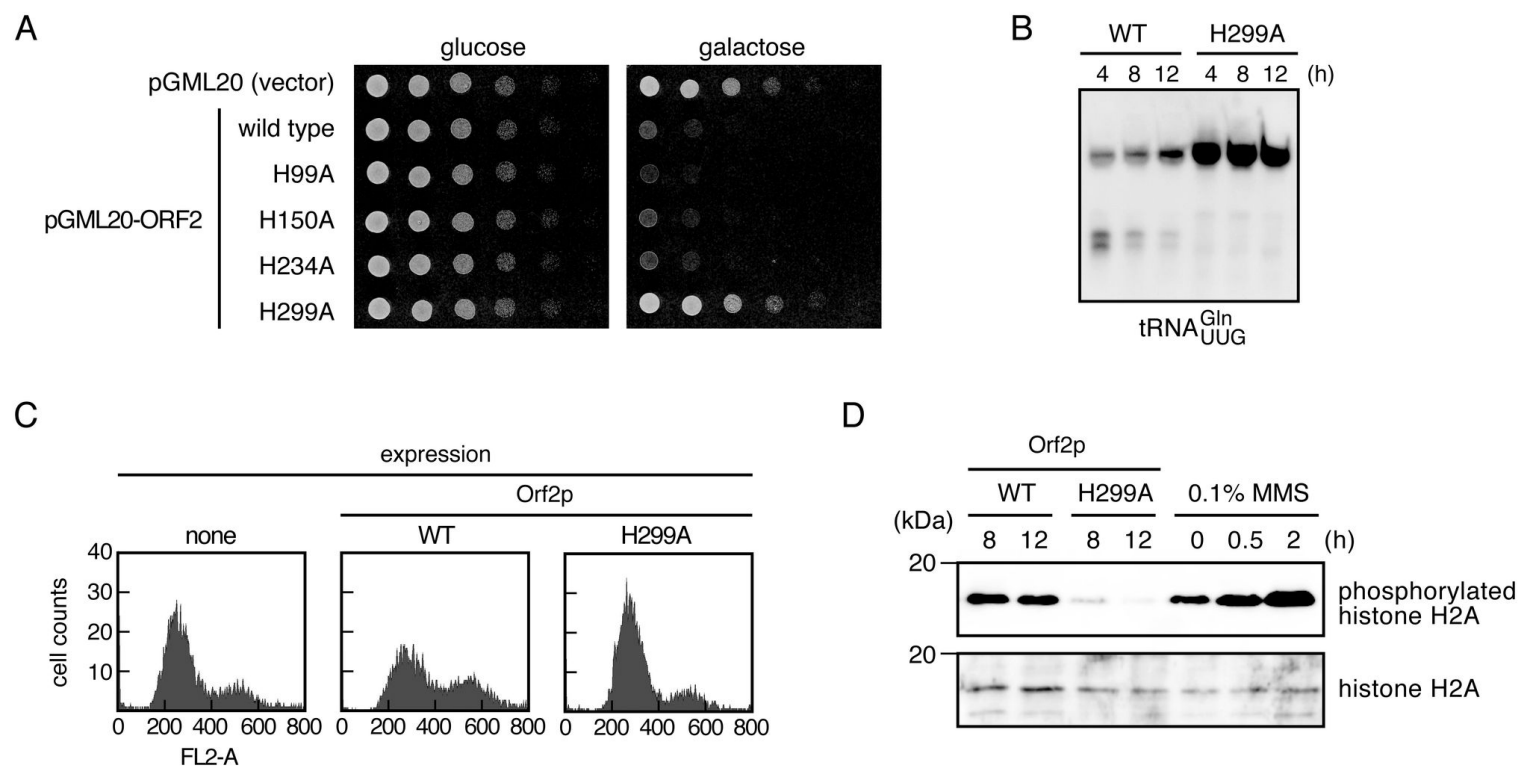
His299 of Orf2p is responsible for tRNA cleavage and DNA damage induction. (A) Four Orf2p mutants, in which each of the His residues was replaced with Ala, were expressed by a high-copy plasmid in TM142 cells, and the viability of these transformants was compared. Orf2p-H299A completely abolished growth impairment. (B) Total tRNA was prepared from CG379 cells chromosomally expressing wild-type (WT) Orf2p or Orf2p-H299A at each interval, and tRNA^Gln^_mcm5s2UUG_ cleavage was observed using northern hybridization. Modification of the first letter of tRNA^Gln^_mcm5s2UUG_ was omitted for simplicity of labeling. Orf2p-H299A did not cleave tRNA^Gln^_mcm5s2UUG_, showing that His299 is indispensable for tRNA cleavage. (C) Wild-type or mutant Orf2p were expressed for 12 hours as shown in (B), and then cells were collected and status of cell cycle was analyzed with fluorescence-activated cell sorting. The cell number is shown on the vertical axis, and the horizontal axis indicates the fluorescence intensity of nuclei stained with propidium iodide. Cell cycle progression of cells in which Orf2p-H299A was expressed was not impaired. (D) Phosphorylation of histone in cells expressing wild-type and mutant Orf2p was determined with western blotting at the indicated time points, as shown in (B). A DNA damage response was not induced in cells expressing Orf2p-H299A, as indicated by a lack of phosphorylation.

**Figure 3:**
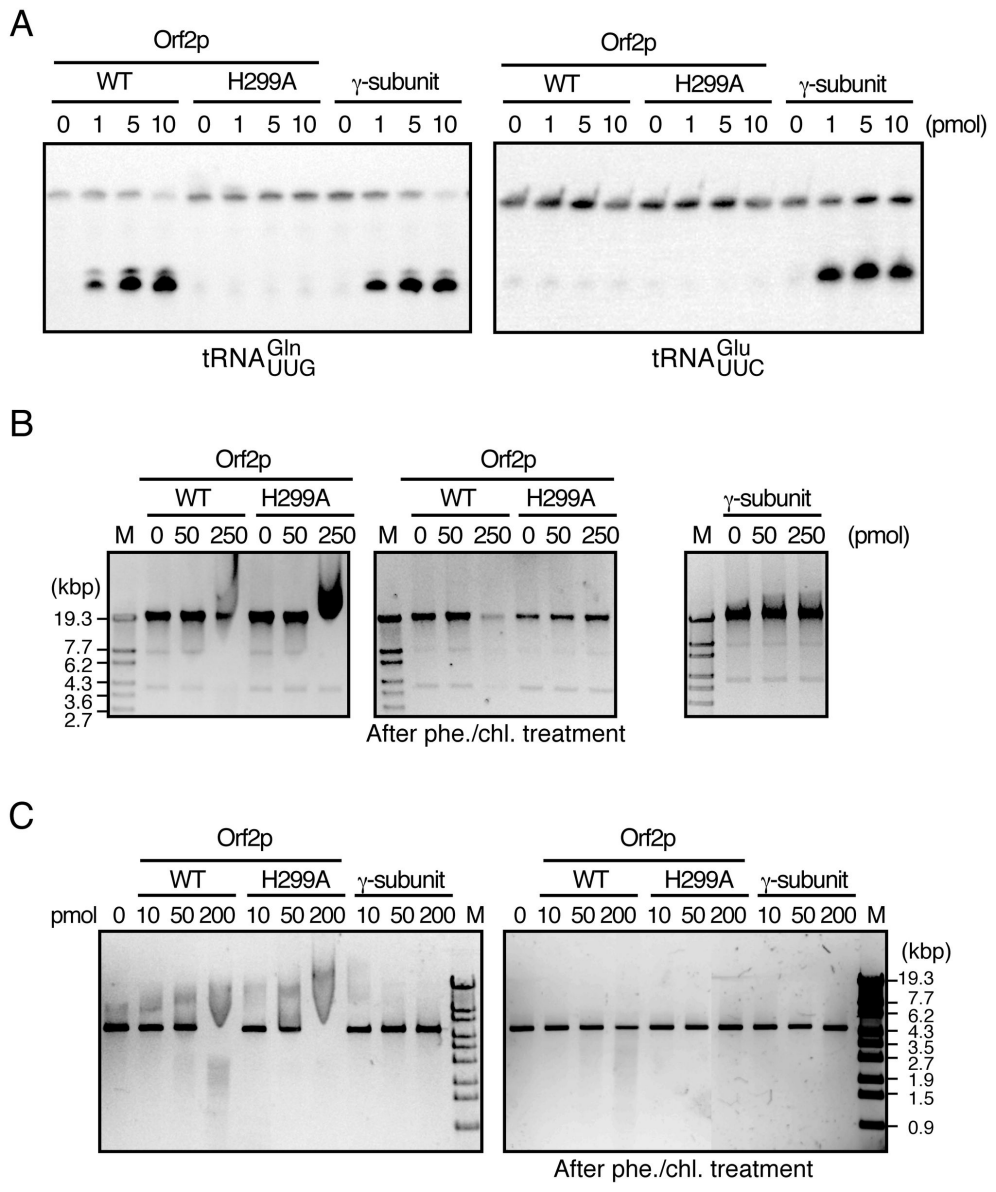
Orf2p cleaves DNA via His299 *in vitro*. (A) Wild-type Orf2p, Orf2p-H299A, and γ-subunit were incubated with total RNA prepared from *S. cerevisiae*. Then, northern hybridization was carried out for the detection of tRNAs, as indicated. Modification of the first letter of tRNA^Glu^_mcm5s2UUC_ and tRNA^Gln^_mcm5s2UUG_ was omitted for simplicity of labeling. Purified Orf2p retained substrate specificity, and Orf2p-H299A lost tRNA cleavage activity. (B) Wild-type (WT) Orf2p, Orf2p-H299A, and γ-subunit were incubated with *Saccharomyces cerevisiae* genomic DNA and subjected to agarose gel electrophoresis. (C) Linearized pGMH10 was used as a substrate instead of genomic DNA. After incubation with Orf2p, DNA was extracted with phenol/chloroform treatment and electrophoresed (middle panel in [B] and right panel in [C]). Linearized λ phage DNA digested with *Sty*I was loaded as a size marker and is indicated as M. Both Orf2p and Orf2p-H299A slowed the electrophoretic mobility of substrate DNA. Moreover, the band became faint after incubation with wild-type Orf2p, but not with its mutant, indicating that DNA cleavage activity of Orf2p depends on His299.

Our findings suggested that Orf2p directly mediates DNA damage, regardless of tRNA cleavage. Next, the requirement of a catalytic residue of Orf2p for the RNase activity was screened to assess whether a mutant Orf2p, which is incapable of tRNA cleavage, still induces the DNA damage response. Previous results have shown that His209 of the γ-subunit is responsible for tRNA cleavage as well as its cytotoxic activity (Figure S1) [21]. Histidine is most commonly used as a general acid-base catalyst in many RNases, including RNase A and T1. The activity of the wild-type Orf2p was the highest between pH 6.5 and pH 7 (Figure S2), which is characteristic of His-catalyzed RNases. Considering these facts, it was likely that Orf2p may also use a His residue(s) as a catalyst. To explore the involvement of the His residue(s) in the catalysis, we replaced 4 His residues, His99, His150, His234, and His299, with Ala (Figure S1) and analyzed the viability of cells expressing the Orf2p mutants. Only the His299 mutation abolished the inhibition of colony formation, and tRNA cleavage was not observed in cells expressing the H299A mutant (Figure 2A and B). While our research was being carried out, Klassen and co-workers reported that His287, which corresponds to His299 according to our numbering, is involved in the catalysis of Orf2p [22]. The recombinant His-tagged wild-type Orf2p, its H299A mutant, and zymocin γ-subunit were purified, and their *in vitro* tRNA cleavage activities were assessed (Figure 3A). tRNA^Gln^_mcm5s2UUG_ but not tRNA^Glu^_mcm5s2UUC_ was cleaved when wild-type Orf2p was incubated with total RNA. On the other hand, Orf2p-H299A mutant exhibited no tRNA cleavage activity. Zymocin γ-subunit cleaved both tRNA^Gln^_mcm5s2UUG_ and tRNA^Glu^_mcm5s2UUC_, which is consistent in the previous result [9]. This result indicates that these recombinant proteins retain substrate specificity, and we did not observe a contamination with other nucleases. Next, the contribution of His299 to cell cycle arrest and DNA damage induction was analyzed. CG379 cells which chromosomally express Orf2p reportedly show no obvious S phase arrest, apparently owing to the lethality of the intracellular expression of Orf2p, which we have discussed in a previous report [16]. However, the entry of cells into the G1 phase was significantly reduced, indicating that wild-type Orf2p blocked cell cycle progression. Conversely, FACS analysis revealed almost no differences between the cells expressing Orf2p-H299A and the control cells (Figure 2C). Moreover, histone H2A phosphorylation was not induced in the cells expressing Orf2p-H299A (Figure 2D).

### Orf2p has DNA cleaving activity

We further investigated the possibility that Orf2p directly interacts with the genomic DNA of sensitive yeast cells. Purified Orf2p and its catalytic mutant were incubated with the chromosomal DNA prepared from *S. cerevisiae* and subjected to agarose gel electrophoresis. Intriguingly, a mobility shift was observed when genomic DNA was incubated with both wild-type Orf2p and Orf2p-H299A (Figure 3B). Conversely, no DNA band shift was observed when γ-subunit was used instead. The band shift disappeared after phenol/chloroform extraction, indicating that Orf2p interacts with genomic DNA, resulting in decreased mobility. Moreover, the band of the genomic DNA with wild-type Orf2p, but not Orf2p-H299A, became faint after phenol/chloroform extraction, suggesting that DNA cleavage occurs through wild-type Orf2p. We further tested this possibility using a linearized plasmid, expecting that the band shift effect and DNA degradation would be observed more clearly compared with that in assays using genomic DNA. A band shift was detected with an even smaller amount of Orf2p compared with that of genomic DNA (Figure 3C). In addition, a smear pattern was observed when only the wild-type Orf2p was used for incubation. This result indicates that DNA degradation did in fact occur. Taking these results together, we concluded that Orf2p interacts with and degrades DNA, and cleaves tRNA by a His299-dependent mechanism.

### Impairment of translation by tRNA cleavage allows the nuclear translocation of Orf2p

**Figure 4:**
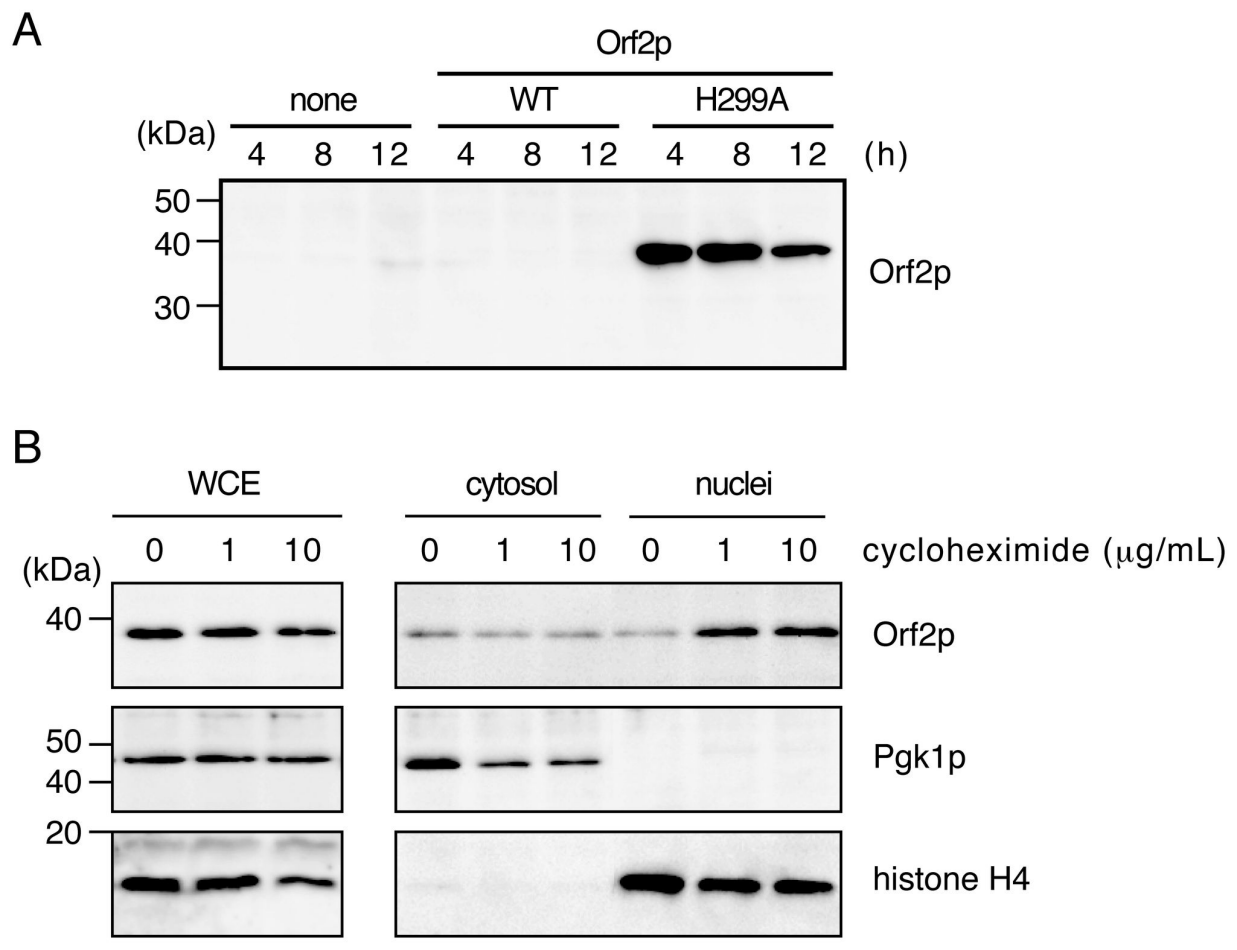
Translation impairment allows nuclear translocation of Orf2p. (A) Wild-type (WT) Orf2p and Orf2p-H299A, which are Flag-tagged at the C-terminus, were expressed in CG379 cells as shown in Figure 2B and detected with western blotting at each time point. Wild-type Orf2p was not detected even after 4 hours of expression. (B) Expression of Orf2p-H299A was induced for 5 hours in cells as shown in (A) followed by 1 hour of cycloheximide treatment as indicated to mimic the translation impairment caused by tRNA cleavage. After nucleus fractionation, Flag-tagged Orf2p-H299A was detected with western blotting. Pgk1p and histone H4 were detected as markers in the cytosol and nucleus, respectively. WCE indicates unfractionated lysate. Band intensities of Orf2p in the nucleus were remarkable with cycloheximide treatment, indicating that the translocation of Orf2p into the nucleus is translation-impairment dependent.

Orf2p needs to translocate into the nucleus to interact with genomic DNA. A previous study has shown that green fluorescent protein (GFP)-tagged Orf2p expression localizes in the cytosol, but not in the nucleus [11]. Nevertheless, GFP tagging may change Orf2p localization. Our previous report has shown that Orf2p expression impairs translation in host cells and induces a similar transcriptional response as the treatment with cycloheximide [16]. This translational inhibition decreases Orf2p expression, thus complicating detection by western blotting (Figure 4A). Orf2p-H299A was then expressed, and the cells were treated with cycloheximide to mimic the translation inhibition induced by tRNA cleavage. After treatment, cell fractionation was carried out, and the localization of Orf2p-H299A in the nucleus was examined by western blotting (Figure 4B). Notably, Orf2p levels in the nucleus increased, although total Orf2p was slightly decreased after cycloheximide treatment. The results suggested that decreased translation activity permits Orf2p to translocate into the nucleus and cleave DNA.

## Discussion

Although PaT reportedly induces DNA fragmentation, the mechanism and correlation with tRNA cleavage had not yet been proven. This study showed that Orf2p, a subunit of PaT responsible for tRNA cleavage, cleaves chromosomal DNA. His299, which is required for tRNA cleavage, is also involved in DNA cleavage. His residues are also found in other DNases, such as DNase I [23]. Our results indicate that tRNA cleavage alone does not induce a DNA damage response, because the histone is not phosphorylated by the expression of D-CRD or γ-subunit. As mentioned, some yeast strains with defective DNA repair systems are more sensitive to PaT and zymocin, suggesting that these repair systems respond to direct DNA cleavage of PaT Orf2p. Conversely, the reason DNA repair is required to compete with zymocin remains unclear. In addition to cleaving tRNA, zymocin reportedly changes the multiple aspects of the physiological status of sensitive yeast cells (including cell cycle progression, phosphorylation status of the polymerase II C-terminal repeat domain region, and mating efficiency [24,25]), which may be involved in the DNA repair process. Furthermore, histone phosphorylation was observed in the cells challenged with zymocin (see Figure 1B).

Notably, tRNA cleavage and the subsequent translation impairment are required for Orf2p translocation into the nucleus, although the mechanism of Orf2p nuclear translocation remains obscure. The integrity of the nuclear membrane may be disrupted by the impairment of translation, thus allowing the translocation. Furthermore, a small amount of Orf2p was detected without cycloheximide treatment (see Figure 4B), suggesting that the translocation of Orf2p into the nucleus is not absolutory dependent on translation impairment. Orf2p is approximately 38 KDa and can pass through the nuclear pore without specific transport proteins such as importin. Alternatively, the translocation of Orf2p might be nuclear localization signal-dependent. The PSORT WWW server (http://psort.hgc.jp/form2.html) suggests that Orf2p contains a nuclear localization signal (Figure S1). We have previously shown that tRNA cleavage is not prolonged and gradually ceases in Orf2p-expressing cells [16]. Conversely, as seen in Figure 1A, histone phosphorylation became pronounced as the experiment progressed. These results suggest that once Orf2p enters the cytoplasm of the sensitive yeast cells, it starts to cleave tRNA. Then, it gradually translocates into the nucleus and cleaves genomic DNA. Klassen has reported that PaT-treated cells show a 2-step cell death process [13]. In the first step, PaT-treated cells maintain viability of approximately 30% for 10 hours. In the second step, the viability drops to nearly 1% during the next 12 hours. The first and second steps might correspond to the tRNA cleavage and DNA cleavage described in this study, respectively.

The mechanism by which both tRNA and DNA are recognized by Orf2p is intriguing in that Orf2p cleaves a specific site in the anticodon loop of target tRNA, whereas DNA cleavage is apparently nonspecific. Although many nucleases have been characterized to date, such an unbalanced specificity of RNA and DNA recognition is uncommon. The ternary structure of tRNA may restrict the cleavage site to the anticodon loop. To understand this mechanism more thoroughly, clarification of whether Orf2p is single-strand DNA specific is needed. The cleavage by γ-subunit depends entirely on the nucleotide modification of the substrate tRNA, whereas the mechanism of Orf2p-mediated cleavage remains obscure. Klassen has reported that a strain lacking *TRM9* is highly tolerant to PaT challenge [13]. *TRM9* encodes tRNA methyltransferase, which is required for the methylation of 5-carboxymethyl-2-thiouridine (cm^5^s^2^U) to generate 5-methoxycarbonylmethyl-2-thiouridine (mcm^5^s^2^U). The fact that *TRM9* deletion only impedes methylation, which is the final step of mcm^5^s^2^U modification [26], suggests that methylation is necessary for the recognition of Orf2p. However, in *vitro* transcribed tRNA^Gln^_UUG_ is still susceptible to Orf2p, indicating that methylation is not absolutely required. Additionally, the DNA cleavage of Orf2p is not apparently influenced by the methylation pattern of plasmid DNA (Figure S3). The substrate recognition of Orf2p is not completely different from that of γ-subunit. tRNA^Glu^_mcm5s2UUC_ and tRNA^Lys^_mcm5s2UUU_ are also reportedly susceptible to Orf2p *in vitro* as well as to tRNA^Gln^_mcm5s2UUG_ [11], indicating that the γ-subunit of zymocin and Orf2p of PaT share tRNA substrates, although their primary target differs. Therefore, further studies are required to understand the substrate recognition mechanism of Orf2p, which will provide insight into the evolutionary relationships between tRNA-targeting RNases.

## Supporting Information

Figure S1 **Comparison of amino acid sequences of zymocin, γ-subunit, and PaT Orf2p.** The underline indicates a signal sequence likely to be required for the secretion from toxin-producing cells, and these sequences were removed in this study. The open box in the middle of the Orf2p sequence is a nuclear localization signal predicted by PSORT. Arrowhead indicates catalytic His residues of the γ-subunit that have been reported previously. The 4 histidine residues indicated by asterisk were replaced by alanine in Orf2p.
(TIF)

Figure S2 **pH dependence of the tRNA cleavage activity of Orf2p.** Total RNA prepared from *Saccharomyces cerevisiae* was incubated with Orf2p in the indicated buffer conditions, and then cleavage efficiency was evaluated with northern hybridization. Transfer RNA cleavage activity of Orf2p was highest at pH7.0, suggesting that His residue is involved in the catalysis.
(TIF)

Figure S3 **Methylation pattern does not influence DNA cleavage *in vitro*.** The pGMH10 plasmid was prepared from *Escherichia coli* K-12 JM109 and GM119 which is defective in two methylases (*dcm*- and *dam*-), and digested with *Sal*I. The linearized DNA was incubated with wild-type (WT) Orf2p and Orf2p-H299A, and applied to an agarose gel. No difference of the susceptibility of these plasmids to Orf2p was observed.
(TIF)
