# Supplementary figures and images for: Evidence for DNA Cleavage Caused Directly by a transfer RNA-Targeting Toxin
Source: PLoS One. 2013 Sep 17;8(9):e75512. doi: 10.1371/journal.pone.0075512 (PMC3775755; doi:10.1371/journal.pone.0075512)

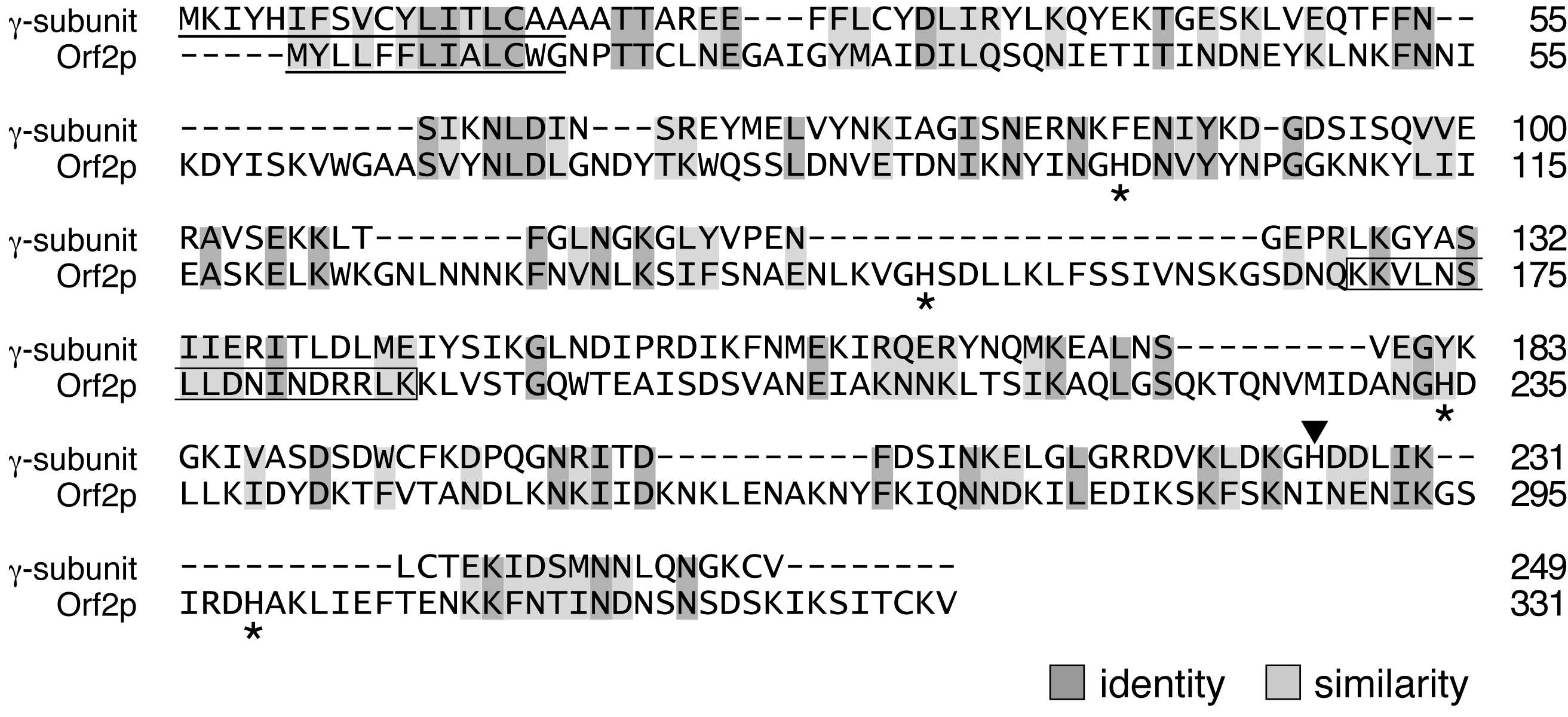

Supplement: Figure S1 — Comparison of amino acid sequences of zymocin, γ-subunit, and PaT Orf2p. The underline indicates a signal sequence likely to be required for the secretion from toxin-producing cells, and these sequences were removed in this study. The open box in the middle of the Orf2p sequence is a nuclear localization signal predicted by PSORT. Arrowhead indicates catalytic His residues of the γ-subunit that have been reported previously. The 4 histidine residues indicated by asterisk were replaced by alanine in Orf2p. (TIF) [file pone.0075512.s001.tif]

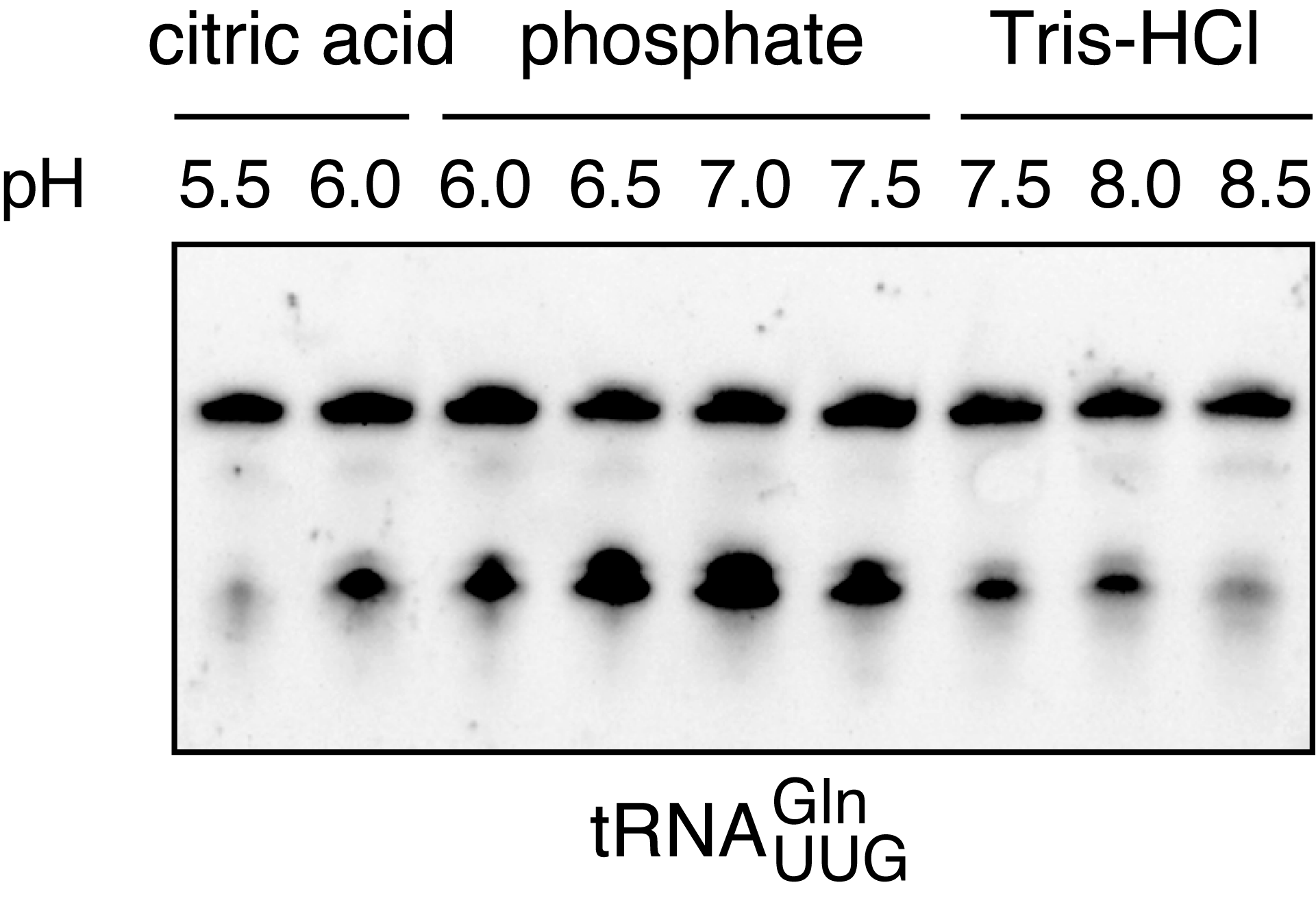

Supplement: Figure S2 — pH dependence of the tRNA cleavage activity of Orf2p. Total RNA prepared from Saccharomyces cerevisiae was incubated with Orf2p in the indicated buffer conditions, and then cleavage efficiency was evaluated with northern hybridization. Transfer RNA cleavage activity of Orf2p was highest at pH7.0, suggesting that His residue is involved in the catalysis. (TIF) [file pone.0075512.s002.tif]

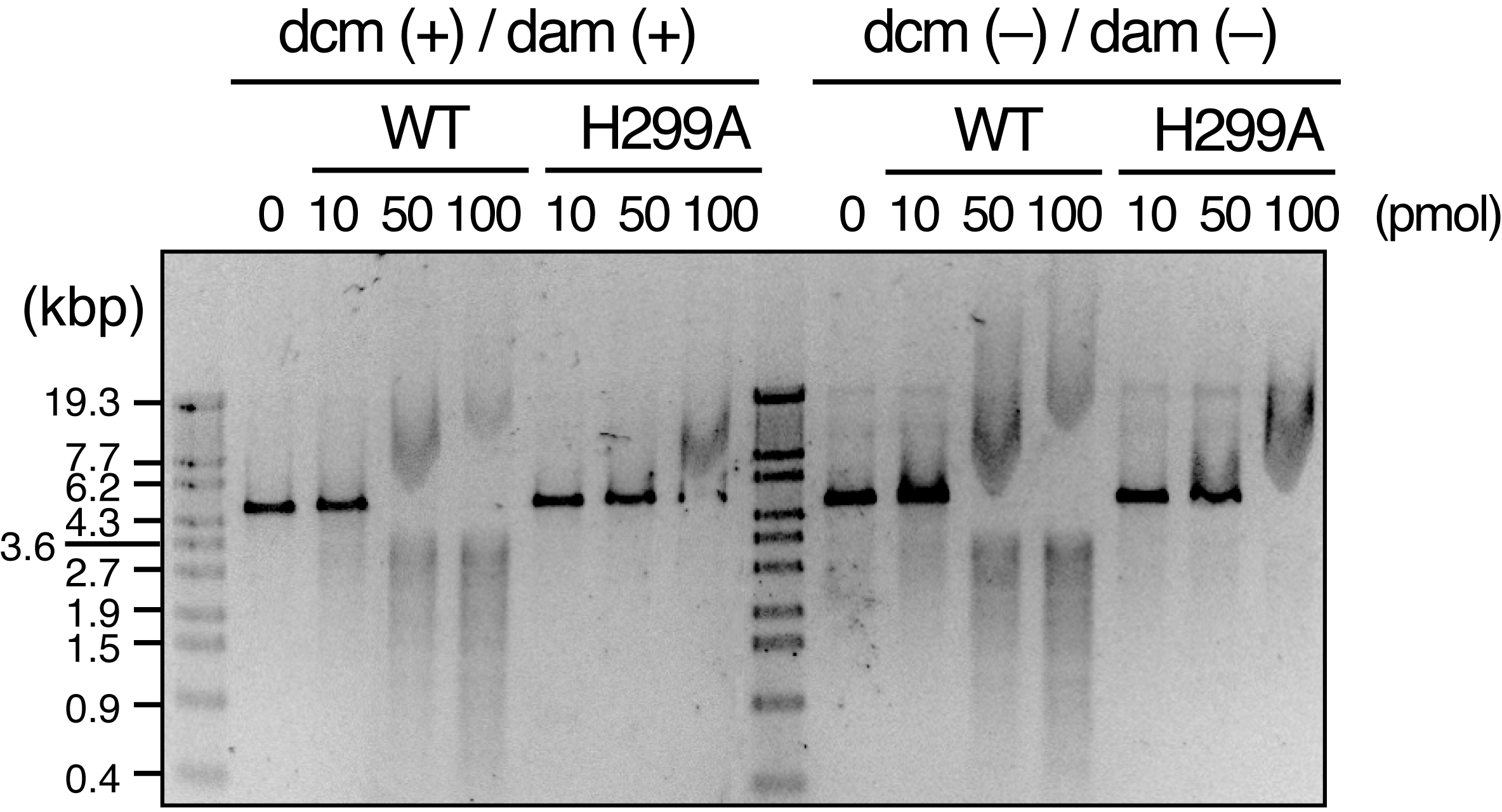

Supplement: Figure S3 — Methylation pattern does not influence DNA cleavage in vitro. The pGMH10 plasmid was prepared from Escherichia coli K-12 JM109 and GM119 which is defective in two methylases (dcm- and dam-), and digested with SalI. The linearized DNA was incubated with wild-type (WT) Orf2p and Orf2p-H299A, and applied to an agarose gel. No difference of the susceptibility of these plasmids to Orf2p was observed. (TIF) [file pone.0075512.s003.tif]
